# A rare case of hairy leukoplakia in a young immunocompetent patient

**DOI:** 10.4317/jced.62391

**Published:** 2025-03-01

**Authors:** Ludimila Lemes Moura, Victor Villatoro Carrapato, Marcia Mirolde Magno de Carvalho Santos, Paulo Sérgio da Silva Santos

**Affiliations:** 1 Department of Surgery, Stomatology, Pathology and Radiology, Bauru School of Dentistry, University of São Paulo, Bauru, SP, Brazil; 2 Ribeirao Preto Medical School, University of São Paulo, Ribeirão Preto, Brazil; 3 Private Practice

## Abstract

We present a case of oral hairy leukoplakia (OHL) in a male, 21-year-old immunocompetent patient. The patient had white, asymptomatic plaques located bilaterally on the tongue margin. The patient noticed the signs days after burning his tongue with hot food, which triggered the investigation. The case was treated initially as candidiasis and frictional keratosis. Exfoliative cytology indicated the presence of actinomycetes in the lesions, and a blood count suggested mild leukopenia (3,910/mm³; reference value 4,500 - 11,000/mm³). Photodynamic therapy and antiseptic mouthwash were used, with no improvement in the condition. The diagnosis of OHL had concluded after an incisional biopsy and immunohistochemical examination for anti-EBV. The patient’s blood and serological tests showed no noteworthy changes. Cases of hairy leukoplakia in immunocompetent patients had founded in the literature and suggest that when this occurs, patients often have some comorbidity, such as hypertension, or use medications such as antihypertensives and steroid inhalers. However, the patient in the present case did not any of these conditions, which made the diagnosis challenging. In any case, it is always important to investigate the medical history and immune status of a patient diagnosed with OHL.

** Key words:**Oral hairy leukoplakia, Epstein-Barr virus, Immunocompetent.

## Introduction

Oral hairy leukoplakia (OHL) is a lesion related to the Epstein-Bar virus (EBV) (1). Over the years, the occurrence of this lesion has been correlated with human immunodeficiency virus (HIV)/AIDS patients, even indicating a compromised immune status in these patients (2,3).

Recently, the OHL has been reported in transplant patients who use immunosuppressive medications, and some rare cases have been reported in immunocompetent patients with no history of using any medication (4,5).

The lesion appears as a white plaque, well delimited, with a corrugated or hair like surface, not detachable, commonly located on the lateral border of the tongue unilaterally or bilaterally. The diagnosis is made based on histopathological analysis of the lesion, involving immunohistochemical tests that confirm the presence of the virus (4,6).

Therefore, we aimed to report a case of oral hairy leukoplakia in an immunocompetent patient to contribute to the understanding of the epidemiology and behavior of this lesion.

## Case Report

A 21-year-old male patient presented complaining of white plaques on the tongue. The medical history revealed that the patient had clinical manifestations suggestive of mononucleosis for approximately two months, and then, about a week before the lesions appeared, he reported that he had burned his mouth with food. The patient was initially diagnosed with candidiasis and then with frictional keratosis. He administered acyclovir, fluconazole, nystatin, and Oncilom®-A Orabase (triamcinolone acetonide) without improvement.

**Figure 1:**
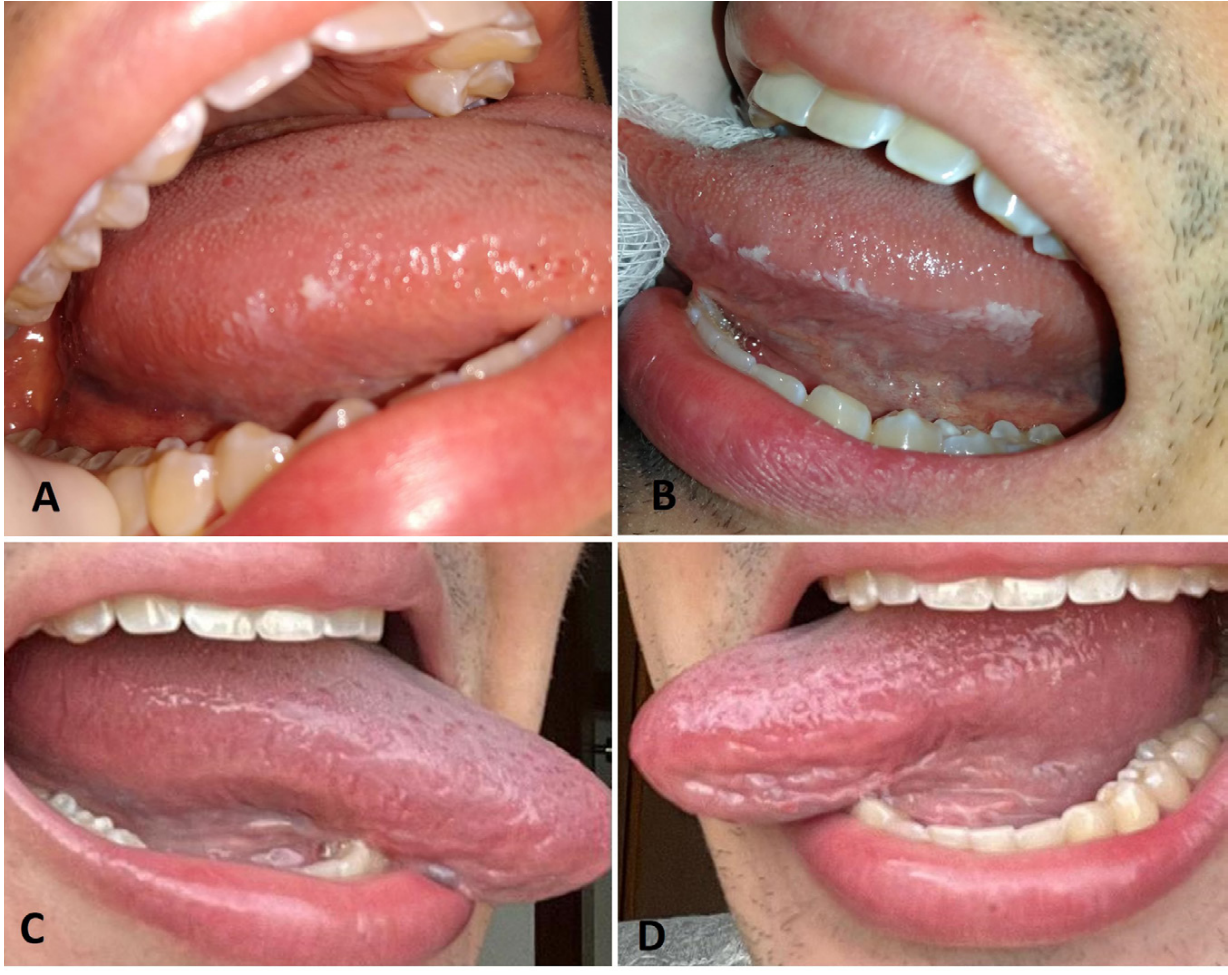
OHL in a 21-year-old man immunocompetent. A,B: showed a removable white curd-like plaque accompanied by a thick, corrugated, asymptomatic white plaque, bilaterally on the lateral border of the tongue. C,D: Showed the lateral border of the patient’s tongue with a normal appearance.

Extraoral examination indicated lymphadenopathy of the submandibular lymph nodes. Intraoral examination revealed white patches on the bilateral border of the tongue. They were asymptomatic and non-detachable (Fig. 1). Relationship with the trauma factor not observed. The exfoliative cytology of the lesions showed the presence of actinomyces bacteria. A full blood count revealed mild leukopenia (3,910/mm³; reference value 4,500 - 11,000/mm³). The patient was screened for HIV, syphilis, hepatitis B and C, and in all cases the result was non-reactive. In addition, immunity tests were performed, such as natural killer cell count, CH50, CD4, CD8, IgA, IgE, T and B lymphocytes, among others, all of which indicate a healthy immune system.

**Figure 2:**
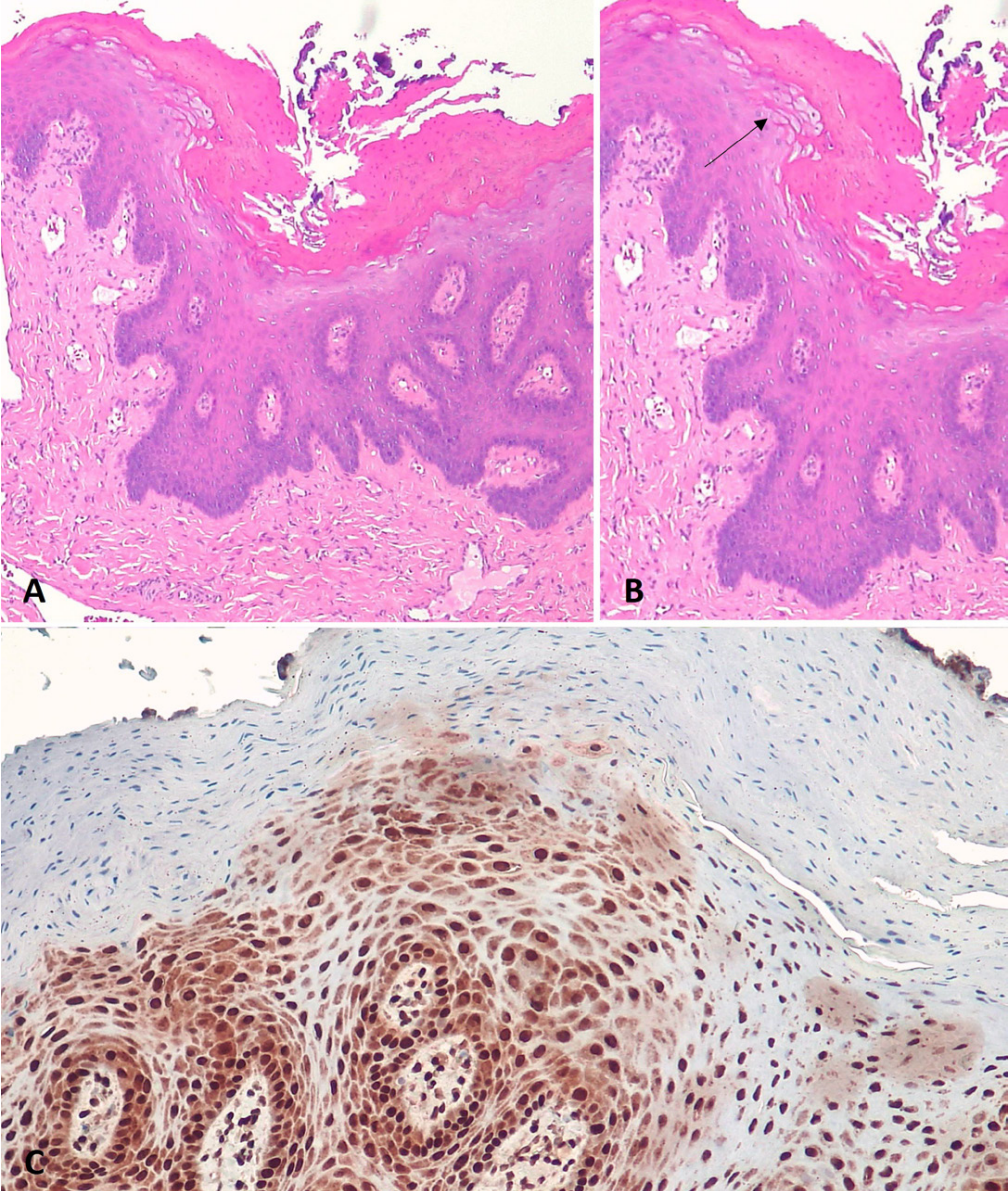
A,B: Hyperparakeratinized stratified squamous epithelium with “balloon cells”, arrowhead. C: Epstein-Barr virus (EBV) in situ hybridization positive in epithelial cell nuclei.

Antimicrobial photodynamic therapy. The initial management was photodynamic therapy on the lesions, there were four sessions with a 5-day interval between sessions, the dye used was methylene blue (0.01%), activated with a visible red laser (660nm), *P*=100mW, E=9J, spot technique with 1 cm between the points on the lateral borders of the tongue. Dentalclean Detox Pro® mouthwash was prescripted twice a day, for fifteen days. After three months, the lesions did not regress. So, an incisional biopsy was performed. Anatomopathological examination revealed the presence of epithelial hyperplasia and extensive hyperparakeratosis with bacterial colonies on the surface. In the squamous epithelium, “ballooned” cells and nuclei with chromatin condensation in the “beading” pattern of the nuclear membrane were observed. These data associated with the positive immunohistochemical examination for anti-EBV in the cells resulted in the diagnosis of OHL, (Fig. 2).

The patient was referred for a review of the medical history, serological tests, immunity investigation, and any noteworthy changes. The patient used Acyclovir for three weeks and, after two months, there was total regression of the lesions.

## Discussion

OHL in a patient with no known systemic immunosuppression suggests serologic investigation of HIV (2,6,7). In the case presented, the patient had an episode suggestive of mononucleosis two months before the appearance of the lesions, which indicates the infection by EVB. Serology for HIV type 1 and type 2 and Hepatitis B e C were non-reactive. The patient’s blood count did not indicate significant changes, other than a small leukopenia. Although hairy leukoplakia is more common in immunosuppressed patients, such as those with HIV, patients with this oral lesion are not always carriers of the virus HIV.

The diagnosis of OHL in a young patient without comorbidities, like in this case report can be challenging. OHL is considered rare in immunocompetent patients, but recently, some cases were reported (4,7,8). The study by (8) *et al*., (2021) reported 45 cases of immunocompetent patients with OHL and the largest of them between the sixth and eighth decades of life (82.2%), age range like the cases of the study by (7) *et al*., (2021). In general, patients had at least one comorbidity such as hypertension (53.3%), hyperlipidemia (42.2%), or chronic respiratory conditions (33.3%) and using some continuous medication such as antihypertensive drugs (21.0%), steroid inhalers (14.6%), and cholesterol-lowering drugs (11.0%) (8). (4) *et al*., 2021) refer to an 18-year-old man, the same profile as our patient, who was using corticosteroids daily due to palate surgery. There is a case of hairy leukoplakia in a 9-year-old child with a history of heart murmur and the use of nasal spray for four months (5). Our patient were not using any medication.

The most common region of occurrence of OHL is in the margin of the tongue (4,7,8) like in our patients. Our patient received the presumptive diagnosis of frictional hyperkeratosis and candidiasis from different professionals he visited. The differential diagnosis most frequently reported in other studies was frictional keratosis, and due to the age group with the highest occurrence of lesions (sixth to the eighth decade of life), dysplasia and carcinoma were also commonly considered.

The characteristics observed in the anatomopathological examination of OHL are non-specific (6). Therefore, to confirm the diagnosis of OHL, it is necessary to verify the presence of the EBV in the cells through the In Situ Hybridization to EBV and LMP1 Immunohistochemistry (9,10) which was the test used to complement the diagnosis of present case.

There is no specific treatment for hairy leukoplakia. The use of antivirals such as Acyclovir is recommended in cases where there is pain (11). There is also no evidence of malignancy (8,11).

The occurrence of OHL is not limited to patients with some immunosuppressive factor. The present case reinforces that the identification of this oral lesion cannot be directly associated with HIV infection. However, upon verifying the presence of this alteration, we emphasize that a careful investigation of the patient’s medical history and serological tests must be carried out.

## Data Availability

The datasets used and/or analyzed during the current study are available from the corresponding author.

## References

[1] Khammissa RA, Fourie J, Chandran R, Lemmer J, Feller L (2016). Epstein-Barr Virus and Its Association with Oral Hairy Leukoplakia: A Short Review. Int J Dent.

[2] Indrastiti RK, Wardhany II, Soegyanto AI (2020). Oral manifestations of HIV: Can they be an indicator of disease severity? (A systematic review). Oral Dis.

[3] Lustosa de Souza BK, Faé DS, Lemos CAA, Verner FS, Machado RA, Ortega RM (2023). Associated oral manifestations with HIV southeastern Brazilian patients on antiretroviral therapy. Braz J Otorhinolaryngol.

[4] Shanahan D, Cowie R, Rogers H, Staines K (2018). Oral hairy leukoplakia in healthy immunocompetent patients: a small case series. Oral Maxillofac Surg.

[5] Costa FH, Costa V, León JE, Anbinder AL, Ribeiro-Silva A, Kaminagakura E (2020). Oral hairy leukoplakia in a child using a corticosteroid nasal spray. Pediatric dermatology.

[6] Flores-Hidalgo A, Lim SO, Curran AE, Padilla RJ, Murrah V (2018). Considerations in the diagnosis of oral hairy leukoplakia-an institutional experience. Oral Surg Oral Med Oral Pathol Oral Radiol.

[7] Alramadhan SA, Bhattacharyya I, Cohen DM, Islam MN (2021). Oral Hairy Leukoplakia in Immunocompetent Patients Revisited with Literature Review. Head and neck pathology.

[8] Almazyad A, Alabdulaaly L, Noonan V, Woo SB (2021). Oral hairy leukoplakia: a series of 45 cases in immunocompetent patients. Oral Surg Oral Med Oral Pathol Oral Radiol.

[9] Gulley ML (2021). Molecular diagnosis of Epstein-Barr virus-related diseases. The Journal of molecular diagnostics: JMD.

[10] Martins LL, Rosseto JHF, Andrade NS, Franco JB, Braz-Silva PH, Ortega KL (2017). Diagnosis of Oral Hairy Leukoplakia: The Importance of EBV In Situ Hybridization. Int J Dent.

[11] Greenspan JS, Greenspan D, Webster-Cyriaque J (2016). Hairy leukoplakia; lessons learned: 30-plus years. Oral diseases.

